# Regional trends in childhood tuberculosis case notification and treatment outcomes: Tanzania, 2018–2023

**DOI:** 10.1016/j.jctube.2026.100640

**Published:** 2026-07-30

**Authors:** Evance N.Mgeyi, Florence S.Kalabamu, Onduru G.Onduru, Kisonga M.Riziki, Robert F.Balama, Galus A.Sililo, Paul Shimba, John Kwesiga, Ainembabazi Rose, Solomon T.Wafula, Deo Nsubuga, Derrick Kimuli

**Affiliations:** a Faculty of Science and Technology, Department of Health Sciences, Cavendish University Uganda, Kampala, Uganda; b National Tuberculosis & Leprosy Programme (NTLP), Ministry of Health, The United Republic of Tanzania, Dodoma, Tanzania; c Department of Disease Control and Environmental Health, Makerere University School of Public Health, Kampala, Uganda; d Department of Pediatrics & Child Health, Kairuki University, Dar es Salaam, Tanzania; e Department of Community Health, Kabale University, Kabale, Uganda; f Department of Programs, Baylor College of Medicine Children's Foundation, Mwanza, Tanzania; g Department of Life Sciences and Bio-Engineering, Nelson Mandela African Institution of Science and Technology, Tanzania

**Keywords:** Childhood TB, HIV, Treatment Outcomes, Tanzania, TB notification

## Abstract

**Background:**

Globally, one-in-two childhood tuberculosis (TB) case remains undiagnosed, and TB remains a leading cause of death among children under 15 years despite favorable treatment outcomes. This study assessed trends in case notification and outcomes among children with TB across ten regions of Tanzania.

**Methods:**

We conducted a retrospective cross-sectional study using data from Tanzania's national electronic TB and Leprosy System for children diagnosed between 2018 and 2023. Mann-Kendall Tests were used to assess the existence of monotonic trends in TB notifications, and distribution of outcomes was analyzed using Wilcoxon rank-sum test or Pearson's Chi-squared test, and binary logistic regression at a 5% significance level.

**Results:**

25,449 childhood TB cases were notified over six-year period, with 14.4% (*n* = 3667) HIV/TB co-infected. Case notifications increased from 2688 in 2018 to a peak of 5421 in 2022 then declined to 4493 in 2023 (*p* = 0.047). Significant differences in unfavorable treatment outcomes were observed between pre- and post-COVID-19 periods (OR 0.52, 95% CI 0.44–0.60). TB/HIV co-infection cases declined from 28.5% (*n* = 766) in 2018 to 8.9% (*n* = 400) in 2023 (*p* = 0.002), while HIV-negative cases rose markedly (*p* = 0.027). Overall, 97.2% of children achieved favorable outcomes. Unfavorable outcomes were common among children who were HIV/TB co-infected, under five years, and 10–15 years.

**Conclusion:**

Childhood TB case notifications increased significantly from 2018 to 2023, indicating improved detection, though with notable regional and HIV-related disparities. Treatment outcomes were generally favorable. HIV-positive children and older age groups faced higher risks of unfavorable outcomes. Targeted interventions are needed to address disparities in case detection and treatment success.

## List of abbreviations

**Table:** 

ART	Antiretroviral Therapy
CI	Confidence Interval
COVID-19	Coronavirus Disease 2019
CTC	Care and Treatment Clinic
eTLS	Electronic TB and Leprosy System
HIV	Human Immunodeficiency Virus
IPD	Inpatient Department
LTFU	Lost to Follow-Up
MOH	Ministry of Health
NaTHREC	National Health Research Ethics Committee
NCD	Non-Communicable Disease
NIMR	National Institute for Medical Research
NTLP	National Tuberculosis and Leprosy Programme
OR	Odds Ratio
PMTCT	Prevention of Mother-to-Child Transmission
RCHS	Reproductive and Child Health Services
SSA	Sub-Saharan Africa
TB	Tuberculosis
THIS	Tanzania HIV Impact Survey
TPT	Tuberculosis Preventive Therapy

## 1 Background

Despite being preventable, curable, and with multiple technological advances, childhood Tuberculosis [TB] remains a major global public health challenge. TB consistently ranks among the leading infectious causes of childhood mortality. Childhood TB is the leading cause of death among HIV-positive children and the second among HIV-negative children [1]. In 2024, an estimated 10.7 million people fell ill with TB worldwide, of whom 1.2 million were children under 15 years, contributing to at least 10% of global incident TB cases [2].

However, the majority of childhood TB cases go undiagnosed due to challenges related to the diagnosis of TB among children [3], [4]. Moreover, of those who are diagnosed and treated, children account for a substantial proportion of deaths. In 2024, approximately 1.23 million people died from TB, of these, about 174,300 were children, representing roughly 14% of all TB deaths globally [2]. Moreover, HIV exacerbates the control and management of TB among children. Recent evidence from a large systematic review indicated a more than 15% childhood TB-related mortality among HIV-TB co-infected children [5].

Sub-Saharan Africa (SSA), the nexus of HIV, health system challenges, poverty, and other factors, accounts for nearly half of the estimated global TB cases among children [[6], [7], [8]]. In addition, disruptions in health system processes, such as pandemics [9], [10] and diagnostic challenges [11], further exacerbate the already substantial childhood TB burden and impede progress toward case detection and treatment targets in the region. In the United Republic of Tanzania, despite programmatic advances, it still struggles with an increasing TB prevalence among children [12]. A Joint External Programme Review and National Tuberculosis and Leprosy Programme (NTLP) reported that childhood TB case notifications represented approximately 14–16% of all-age TB notifications annually in Tanzania, suggesting persistent under-detection of TB among children relative to expected pediatric disease burden [13]. This fell short of the targets set for the National Strategic Plan (NSP 2015–2020), which aimed to increase TB case notifications and detection indicators by strengthening routine case reporting and raising the percentage of notified cases, including childhood TB, by 2020 [14]. The ten regions of Njombe, Iringa, Mbeya, Songwe, Kagera, Tabora, Shinyanga, Mara, Ruvuma, and Geita contribute the highest burden of TB in the country [15]. This study examines a 6-year (2018–2023) trend in childhood TB notification rates and treatment outcomes over time by HIV status with consideration of the effect of the COVID-19 period. The 2018–2023 period encompasses key programmatic transitions: the rollout of Xpert MTB/RIF diagnostics, implementation of NSP V (2015–2020) and launch of NSP VI (2020–2025), scale-up of pediatric TB algorithms, and the COVID-19 pandemic, making this period particularly instructive for evaluating programmatic impacts on case detection and outcomes. Understanding these trends is a key lever for guiding improvements in pediatric TB screening, early diagnosis, and integrated TB/HIV services.

## 2 Methods

### 2.1 Study design

The study adopted a cross-sectional retrospective design using secondary data of children under 15 years (2018–2023) retrieved from the National TB and Leprosy Register of Tanzania. This electronic register collects information on all new TB cases and related demographic, treatment-related, and HIV comorbidity for all age groups. The 2018–2023 period was selected as it encompasses NSP V implementation (2015–2020), the launch of NSP VI (2020–2025), progressive Xpert rollout, the COVID-19 pandemic, and the most recent complete treatment cohort at data extraction, enabling evaluation of programmatic and pandemic effects on pediatric TB.

### 2.2 Study setting

The records were extracted for the top ten study regions were selected a priori based on their highest HIV prevalence as per the Tanzania HIV Impact Survey (THIS) in 2022–2023, before data extraction: [Njombe (12.7%), Iringa (11.1%), Mbeya (9.6%), Songwe (6.0%), Kagera (5.7%), Tabora (5.6%), Shinyanga (5.6%), Mara (5.0%), Ruvuma (4.9%) and Geita (4.9%)] [15]. These regions also correspond to areas with the highest TB notification rates nationally (NTLP Annual Report 2023), reflecting the well-documented co-distribution of HIV and TB burden. Tanzania, located in East Africa with a population of approximately 61 million, is classified as a high TB burden country by the World Health Organization. According to the 2022 Tanzania National Population Census, the combined population of the ten study regions is approximately 21.6 million, representing roughly 35.4% of Tanzania's total population. The country's TB program operates under the NTLP, which coordinates diagnosis, treatment, and surveillance activities through a network of public and private health facilities. Diagnostic capacity varied across regions. All ten regions had sputum smear microscopy at district level. Xpert MTB/RIF was progressively rolled out from regional referral hospitals during the study period, with coverage expanding from urban-only sites in 2018 to broader district-level availability by 2022–2023. TB culture facilities remain centralized at the national reference laboratory.

### 2.3 Study population

The study population comprised all children under 15 years of age who were diagnosed with TB and registered in the National TB and Leprosy Register during 2018–2023. For comparative analyses, the pre-COVID period was defined as 2018–2019 and the COVID/post-COVID period as 2020–2023, aligned with Tanzania's documented COVID-19 response timeline. This included children with all forms of TB (pulmonary and extra-pulmonary) and all diagnostic categories (bacteriologically confirmed and clinically diagnosed). Adults and children with missing data or unknown HIV status were excluded to allow accurate HIV-stratified analyses. All other eligible registered cases were included.

### 2.4 Sample size

All children diagnosed with TB with known HIV status were considered. No sample size calculation was necessary as all records available were considered.

### 2.5 Data sources and measurement of variables

Secondary data were obtained from the National TB and Leprosy System after securing data utilization authorization from the MOH, Tanzania. Data were extracted using a structured data extraction checklist. The checklist comprised Demographics, Referral to TB services, Methods of Diagnosis, TB Disease Classification, Treatment regimens, HIV Status, TB/HIV Collaborative Services, and Treatment outcomes. The outcome of interest was treatment outcome, categorized by the WHO and the Tanzania NTLP [16], [17], [18], [19]. According to this categorisation, Favorable Treatment Outcomes include cured and completed treatment, while unfavorable treatment outcomes were treatment failures, Loss to follow-up (LTFU), and death. Covariates included HIV status (Positive /Negative), age in years categorized in groups, sex (Male /Female), regions (Njombe, Iringa, Mbeya, Songwe, Kagera, Tabora, Shinyanga, Mara, Ruvuma, and Geita), treatment regimen (2HRZE/10RH, 2RHZE/4RH, 2RHZE/4RH-KID), Disease classification (Pulmonary, extra pulmonary or both) and history of treatment (New case, relapse, treatment after lost to follow up, other).

2RHZE/4RH denotes the standard fixed-dose combination in adult tablet formulation used for older children; 2RHZE/4RH-KID refers to the WHO-recommended dispersible, weight-band optimized pediatric fixed-dose combination introduced into Tanzania's NTLP supply chain during the study period for children unable to swallow standard tablets. The 2HRZE/10RH (12-month continuation) regimen was prescribed for children with severe or complicated TB forms. The 4RH continuation phase was used for uncomplicated pulmonary and extra-pulmonary TB.

Treatment outcomes were defined per WHO/NTLP criteria: Cured: bacteriologically confirmed TB with a negative smear or culture result at end of treatment; Treatment completed: completed a full treatment course without evidence of bacteriological confirmation; Treatment failure: positive smear or culture result at month 5 or later; LTFU: treatment interrupted for ≥2 consecutive months; Death: death from any cause during treatment.

### 2.6 Statistical analysis

Data were extracted from the National Register and cleaned using Microsoft Excel Professional 2021. Data quality procedures comprised: (1) automated range and logic checks against WHO/NTLP permissible value sets; (2) deduplication using unique patient identifiers, name, region, and registration year; (3) cross-validation of outcome denominators against published NTLP aggregate reports; and (4) resolution of missing or inconsistent entries in consultation with NTLP data managers. A sensitivity analysis was conducted by repeating primary trend and outcome analyses after excluding records with missing outcome data and missing regimen data. Findings were not materially altered.

All statistical analyses were performed using R software (version 4.5.1) at a 5% significance level. Descriptive statistics were calculated for all variables: continuous variables were summarized using means and standard deviations (or medians and interquartile ranges for non-normally distributed data), while categorical variables were presented as frequencies and percentages.

The Mann-Kendall test was used to assess monotonic trends in annual TB case notifications over the study period. This non-parametric test was selected because it does not assume normality of the data distribution, making it appropriate for count data that may not follow a normal distribution. The distribution of treatment outcomes across patient characteristics was analyzed using Pearson's chi-squared test (or Fisher's Exact Test as applicable) for categorical variables and the Wilcoxon rank-sum test for continuous variables. Binary logistic regression was performed to identify factors associated with unfavorable treatment outcomes. Given the low prevalence of unfavorable outcomes (2.8%), unadjusted analyses are presented in the main results to avoid overfitting and maintain adequate statistical power. However, adjusted multivariable logistic regression results are provided as supplementary materials for reference. Results are reported as odds ratios (ORs) with 95% confidence intervals (CIs).

## 3 Results

### 3.1 Study profile

**Fig. 1:**
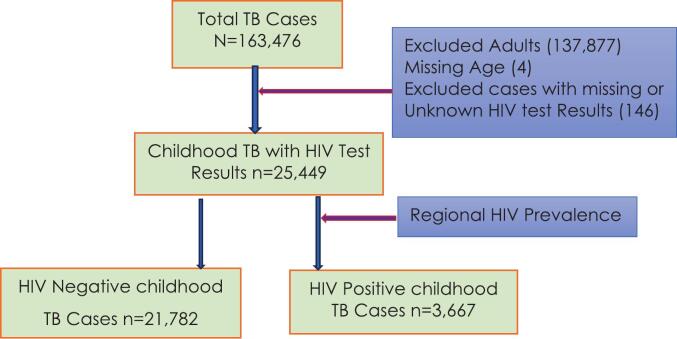
Study Profile of Children Diagnosed and Treated with Childhood TB from 2018 to 2023.

Nationally, Tanzania notified an estimated 525,225 TB cases of all ages between 2018 and 2023 [20], [21], [22], [23], [24], [25] the ten study regions, which notified 163,476 TB cases of all ages over the same period, therefore represent approximately 31.1% of the national TB caseload. After excluding adults (84.3%; 137,877/163,476), missing age data (0.0%; 4/163,476), and cases with unknown HIV status (0.009%; 146/163,476), 15.6% (25,449/163,476) childhood TB cases remained. Of these, *n* = 3667 (14.4%) were HIV co-infected while *n* = 21,782 (85.6%) were HIV-negative (Fig. 1). The pediatric proportion varied by region, from 4.4% in Njombe region to 15.1% in Tabora (see Table 1). All 25,449 registered cases had a documented treatment regimen, indicating near-universal treatment initiation among registered children. The register captures only cases enrolled in treatment; the number of diagnosed children not initiated on therapy prior to registration could not be quantified from this dataset.

**Table 1:** Characteristics of TB cases notified among children in 10 regions of Tanzania (2018–2023).

Characteristic	*N* = 25,449^1^	HIV Negative *N* = 21,782^1^	HIV Positive *N* = 3667^1^	*p*-value^2^
**Age (years)**	**5.7 (4.5)**	**5.4 (4.4)**	**7.4 (4.5)**	**<0.001**
<5	13,047 (51.3%)	11,873 (91.0%)	1174 (9.0%)	
5–9	6325 (24.9%)	5207 (82.3%)	1118 (17.7%)	
10–15	6077 (23.9%)	4702 (77.4%)	1375 (22.6%)	

**Sex**				**0.814**
Female	12,134 (47.7%)	10,379 (85.5%)	1755 (14.5%)	
Male	13,315 (52.3%)	11,403 (85.6%)	1912 (14.4%)	

**Region**				**<0.001**
Geita	3102 (12.2%)	2532 (81.6%)	570 (18.4%)	
Iringa	1381 (5.4%)	1056 (76.5%)	325 (23.5%)	
Kagera	3632 (14.3%)	3335 (91.8%)	297 (8.2%)	
Mara	3661 (14.4%)	3391 (92.6%)	270 (7.4%)	
Mbeya	1472 (5.8%)	1091 (74.1%)	381 (25.9%)	
Njombe	1112 (4.4%)	782 (70.3%)	330 (29.7%)	
Ruvuma	2777 (10.9%)	2592 (93.3%)	185 (6.7%)	
Shinyanga	3596 (14.1%)	3090 (85.9%)	506 (14.1%)	
Songwe	871 (3.4%)	745 (85.5%)	126 (14.5%)	
Tabora	3845 (15.1%)	3168 (82.4%)	677 (17.6%)	

**Year**				**<0.001**
2018	2688 (10.6%)	1922 (71.5%)	766 (28.5%)	
2019	3404 (13.4%)	2670 (78.4%)	734 (21.6%)	
2020	4536 (17.8%)	3840 (84.7%)	696 (15.3%)	
2021	4907 (19.3%)	4380 (89.3%)	527 (10.7%)	
2022	5421 (21.3%)	4877 (90.0%)	544 (10.0%)	
2023	4493 (17.7%)	4093 (91.1%)	400 (8.9%)	

**TB Regimen**				**<0.001**
2HRZE/10RH	477 (1.9%)	435 (91.2%)	42 (8.8%)	
2RHZE/4RH	15,201 (60.4%)	12,728 (83.7%)	2473 (16.3%)	
2RHZE/4RH-KID	9477 (37.7%)	8369 (88.3%)	1108 (11.7%)	
Unknown	294	250	44	

2RHZE/4RH-KID = WHO-recommended weight-band optimized pediatric dispersible fixed-dose combination

-Reporting completeness was assessed for each region-year stratum. All ten regions contributed data for each of the six study years. A sensitivity analysis restricting to regions with complete year-on-year reporting did not materially alter the observed trend in HIV co-infection decline

- The 2HRZE/10RH (12-month continuation) regimen was prescribed for children with severe or complicated TB forms including TB meningitis and osteoarticular TB, per Tanzania NTLP guidelines. The 4RH continuation phase was used for uncomplicated pulmonary and extra-pulmonary TB.

1 Mean (SD); n (%)

2 Wilcoxon rank sum test; Pearson's Chi-squared test

### 3.2 Characteristics of childhood TB cases notified between 2018 and 2023

The overall mean age was 5.7 years (SD ±4.5), with over half of cases (51.2%; 13,055/25,476) occurring in children under five years of age. HIV-positive children were significantly older than their HIV-negative counterparts (mean age 7.4 vs. 5.4 years, *p* < 0.001). HIV positivity increased progressively with age, from 10.8% among children under five years to 22.6% among those aged 10–14 years. Marked regional variation was observed, with higher HIV positivity rates in Njombe (29.7%) and Mbeya (25.9%) compared to less than 10% in Mara and Ruvuma regions. Temporal trends revealed a steady increase in case notifications, peaking at 5421 cases in 2022, while HIV positivity among notified cases declined substantially from 28.5% in 2018 to 8.9% in 2023. The majority of children (60.4%, *n* = 15,369) received the standard 2RHZE/4RH treatment regimen. Regimen distribution differed significantly by HIV status (*p* < 0.001) (Table 1); however, as HIV-positive children were older on average, this pattern more plausibly reflects age- and weight-band-based regimen selection rather than a direct effect of HIV status, and the two factors could not be disentangled without multivariable analysis.

### 3.3 Childhood TB case notifications and outcomes

Overall childhood TB notifications increased significantly from 2688 in 2018 to a peak of 5421 in 2022 before declining slightly to 4493 in 2023 (*p* = 0.047); with statistically significant increases among females (*p* = 0.040), pulmonary TB cases (*p* = 0.027), and new diagnoses (*p* = 0.045). HIV-negative children drove much of this growth, rising from 1922 to 4877 cases (p = 0.027), while HIV-positive cases moved in the opposite direction, declining by nearly 48%, from 766 in 2018 to 400 in 2023 (*p* = 0.002).

**Fig. 2:**
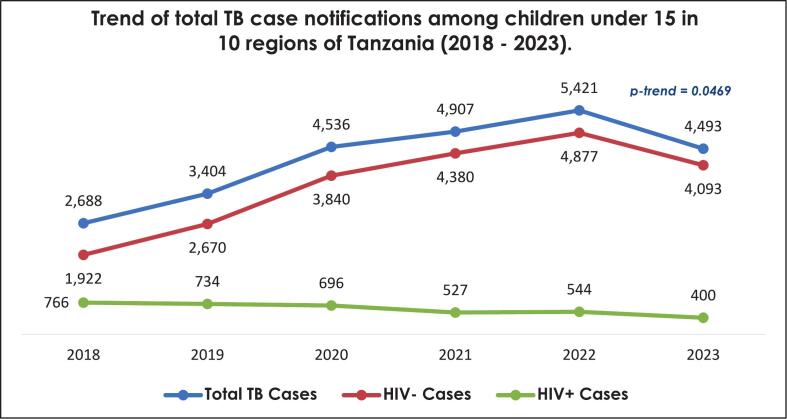

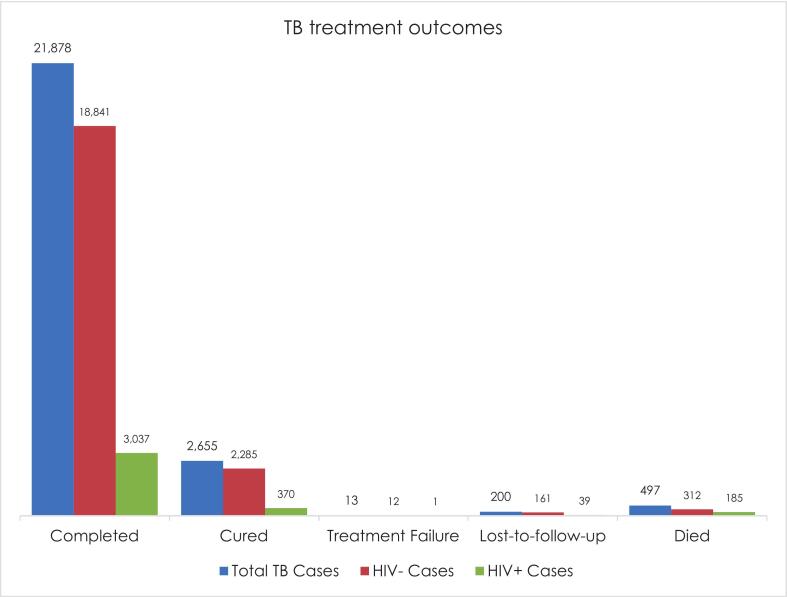
(a) Trend of total TB case notifications among children in 10 regions of Tanzania (2018–2023). Footnote: Incidence rates are not presented as disaggregated regional population denominators by pediatric age group were unavailable. Absolute notifications are presented as the best available programmatic indicator. (b) Childhood TB treatment outcomes among children diagnosed with TB from 2018 to 2023. Footnote: The denominator for treatment outcome analysis is 25,243 after excluding 206 records with missing treatment outcome data; 25,449 reflects the total notified cases.

In contrast, the number of HIV-co-infected childhood TB cases declined steadily from 766 (28.5% of all childhood TB) in 2018 to 400 (8.9%) in 2023 (p = 0.002), whereas notifications among HIV-negative children rose markedly over the same period (p = 0.027) (Fig. 2). Age-group specific TB incidence rates could not be calculated due to the unavailability of disaggregated regional population denominators by single-year age group. Absolute case notifications stratified by age group are presented (Table 1).

The regional decline in TB-HIV co-infection was consistent across most regions, reaching significance in Geita (*p* = 0.021), Iringa (*p* = 0.042), Mbeya (*p* = 0.018), Njombe (*p* = 0.031), Ruvuma (*p* = 0.024), and borderline significance in Shinyanga (*p* = 0.050), with both sexes showing strong downward trends (*p* = 0.003 each). Decreases were especially pronounced in extra-pulmonary TB (*p* < 0.001), pulmonary TB (*p* = 0.007), and new cases (p = 0.002).

Treatment outcomes were excellent overall, with 97.2% (24,533/25,249) of children achieving favorable outcomes (cured or treatment completed) and only 2.8% experiencing unfavorable outcomes (death, loss to follow-up or treatment failure) (Fig. 2). Unfavorable outcomes were significantly more common among HIV-co-infected children (6.2% vs. 2.2% in HIV-negative, p < 0.001), younger children (3.2% under-5 s vs. 2.1% among 5–9 and 2.7% among 10–14 year-olds, p < 0.001), certain regions (highest in Tabora at 5.9%, p < 0.001), and referrals from inpatient departments or care and treatment clinics (6.4%, p < 0.001) (Table 2).

**Table 2:** Childhood TB Treatment outcomes by patient demographic characteristics.

Characteristic	Overall (N = 25,449^1^)	Unfavorable treatment outcomes		p-value^2^
		No [*n* = 24,533^1^]	Yes [*n* = 710^1^]	
**Age (years)**	**5.7 (4.5)**	**5.7 (4.5)**	**5.1 (4.7)**	**<0.001**
< 5	13,047 (51.3%)	12,549 (96.8%)	416 (3.2%)	<0.001
5–9	6325 (24.9%)	6128 (97.9%)	134 (2.1%)	
10–15	6077 (23.9%)	5856 (97.3%)	160 (2.7%)	

**Sex**				**0.214**
Female	12,134 (47.7%)	11,706 (97.3%)	322 (2.7%)	
Males	13,315 (52.3%)	12,827 (97.1%)	388 (2.9%)	

**Region**				**<0.001**
Geita	3102 (12.2%)	3001 (97.1%)	89 (2.9%)	
Iringa	1381 (5.4%)	1317 (95.9%)	57 (4.1%)	
Kagera	3632 (14.3%)	3582 (98.8%)	44 (1.2%)	
Mara	3661 (14.4%)	3454 (97.9%)	75 (2.1%)	
Mbeya	1472 (5.8%)	1415 (96.6%)	50 (3.4%)	
Njombe	1112 (4.4%)	1048 (96.4%)	39 (3.6%)	
Ruvuma	2777 (10.9%)	2747 (99.0%)	29 (1.0%)	
Shinyanga	3596 (14.1%)	3533 (98.3%)	62 (1.7%)	
Songwe	871 (3.4%)	830 (95.4%)	40 (4.6%)	
Tabora	3845 (15.1%)	3606 (94.1%)	225 (5.9%)	

**Year**				**<0.001**
2018	2688 (10.6%)	2516 (95.3%)	125 (4.7%)	
2019	3404 (13.4%)	3222 (95.9%)	139 (4.1%)	
2020	4536 (17.8%)	4381 (96.6%)	154 (3.4%)	
2021	4907 (19.3%)	4783 (97.8%)	107 (2.2%)	
2022	5421 (21.3%)	5279 (98.3%)	93 (1.7%)	
2023	4493 (17.7%)	4352 (97.9%)	92 (2.1%)	

**TB regimen1**				**0.597**
2HRZE/10RH	477 (1.9%)	456 (97.0%)	14 (3.0%)	
2RHZE/4RH	15,201 (60.4%)	14,635 (97.2%)	426 (2.8%)	
2RHZE/4RH-KID	9477 (37.7%)	9180 (97.4%)	247 (2.6%)	
Unknown	294	262	23	

**Disease classification**				**0.114**
Both	151 (0.6%)	141 (94.6%)	8 (5.4%)	
Extra pulmonary	7930 (31.2%)	7643 (97.3%)	209 (2.7%)	
Pulmonary	17,356 (68.2%)	16,737 (97.1%)	493 (2.9%)	
Unknown	12	12	0	

**HIV test results**				**<0.001**
Negative	21,782 (85.6%)	21,126 (97.8%)	485 (2.2%)	
Positive	3667 (14.4%)	3407 (93.8%)	225 (6.2%)	

**Referrals**				**<0.001**
Community	12,066 (47.4%)	11,776 (98.4%)	192 (1.6%)	
CTC	1821 (7.2%)	1725 (95.5%)	82 (4.5%)	
IPD	1514 (5.9%)	1412 (93.6%)	97 (6.4%)	
Others	1146 (4.5%)	1073 (94.9%)	58 (5.1%)	
RCHS	1541 (6.1%)	1487 (96.6%)	52 (3.4%)	
Self	7360 (28.9%)	7059 (96.9%)	229 (3.1%)	

**History of Treatment**				**0.153**
New	25,236 (99.2%)	24,335 (97.2%)	700 (2.8%)	
Other	37 (0.1%)	33 (97.1%)	1 (2.9%)	
Relapse	110 (0.4%)	104 (95.4%)	5 (4.6%)	
LTFU	65 (0.3%)	60 (93.8%)	4 (6.3%)	
Unknown	1	1	0	

2RHZE/4RH-KID = WHO-recommended weight-band optimized pediatric dispersible fixed-dose combination

1 Mean (SD); n (%), 215 participants had no information on treatment outcomes (i.e. missing data).

2 Wilcoxon rank sum test; Pearson's Chi-squared test; Fisher's exact test.

In unadjusted logistic regression, HIV co-infection was the strongest predictor of unfavorable outcomes (OR 2.88, 95% CI 2.44–3.38, p < 0.001). Notification during or after COVID-19 was associated with lower odds (OR 0.52, 95% CI 0.44–0.60, p < 0.001), as was older age — children aged 5–9 years (OR 0.66, 95% CI 0.54–0.80, p < 0.001) and 10–15 years (OR 0.82, 95% CI 0.68–0.99, *p* = 0.040) both showed reduced odds of unfavorable outcomes compared to under-5 s (Table 3).

**Table 3:** Unadjusted odds ratios for unfavorable treatment outcomes (death, LTFU, or treatment failure) among children with TB, Tanzania 2018–2023.

Characteristic	OR	95% CI	p-value
**Age**	**0.97**	**0.95–0.99**	**<0.001**
<5	Ref	Ref	
5–9	0.66	0.54–0.80	<0.001
10–15	0.82	0.68–0.99	0.040

**Sex**			
Female	Ref	Ref	
Male	1.10	0.95–1.28	0.214

**Region**			
Geita	Ref	Ref	
Iringa	1.46	1.04–2.04	0.029
Kagera	0.41	0.29–0.59	<0.001
Mara	0.73	0.54–1.00	0.050
Mbeya	1.19	0.83–1.69	0.329
Njombe	1.25	0.85–1.83	0.245
Ruvuma	0.36	0.23–0.54	<0.001
Shinyanga	0.59	0.42–0.82	0.002
Songwe	1.63	1.10–2.36	0.012
Tabora	2.10	1.64–2.71	<0.001

**Year**	**0.80**	**0.77–0.84**	**<0.001**
Before COVID-19 (2018–2019)	Ref		
During COVID-19 (2020−2023)	0.52	0.44, 0.60	<0.001

**DSTB regimen1**			
2HRZE/10RH	Ref	Ref	
2RHZE/4RH	0.95	0.57–1.71	0.847
2RHZE/4RH-KID	0.88	0.53–1.59	0.636

**Disease classification**			
Both	Ref	Ref	
Extra pulmonary	0.48	0.25–1.08	0.049
Pulmonary	0.52	0.27 Ref 1.16	0.074
HIV test results			
Negative	Ref	Ref	
Positive	2.88	2.44–3.38	<0.001

**Referrals1**			
Community	Ref	Ref	
CTC	2.92	2.23–3.78	<0.001
IPD	4.21	3.27–5.40	<0.001
Others	3.32	2.44–4.45	<0.001
RCHS	2.14	1.56–2.90	<0.001
Self	1.99	1.64–2.42	<0.001

**History of treatment1**			
New	Ref	Ref	
Other	1.05	0.06–4.89	0.959
Relapse	1.67	0.59–3.71	0.264
Treatment after lost to follow up patient	2.32	0.70–5.64	0.105

Abbreviations: CI = Confidence Interval, OR = Odds Ratio

Note: Adjusted estimates not provided due to small samples of negative treatment outcomes.

## 4 Discussion

This study analyzed a six-year (2018–2023) trend of pediatric TB notifications and treatment outcomes across the ten high-burden regions of Tanzania. Childhood TB notifications increased significantly, peaking in 2022, before declining during 2023 (a 67% increase over the study period). HIV co-infection among notified childhood TB cases declined dramatically while notifications among HIV-negative children more than doubled. The declining TB-HIV co-infection rates observed are consistent with national trends. Tanzania's THIS 2022–2023 reports a decline in HIV prevalence from 4.9% (2016–2017) to 4.4% (2022−2023), with Prevention of Mother-to-Child Transmission (PMTCT) coverage exceeding 95%, contributing to reductions in pediatric HIV acquisition and, consequently, TB-HIV co-infection. Treatment outcomes were excellent though HIV-positive children and those under five years experienced unfavorable outcomes. Regional heterogeneity was observed, with the strongest increases in Kagera, Mara, Ruvuma, Tabora, and Songwe regions. NTLP programme records indicate these regions implemented intensive community-based active case-finding campaigns, received donor-supported quality-improvement initiatives, and were prioritized for earlier district-level Xpert MTB/RIF rollout, whereas comparator districts with smaller increases experienced later diagnostic scale-up and less intensive partner support. These district-level differences offer transferable lessons on which specific interventions most effectively improved pediatric case detection. Unlike global trends showing substantial COVID-19 disruptions, Tanzania maintained stable or increasing notifications throughout the pandemic period (2020−2022), with the decline occurring post-pandemic in 2023.

Case notifications increased significantly, peaking in 2022 (a 102% increase). With the country's approximately 3% annual population growth [25], [26], this may have partly contributed to a roughly 18% increase over six years. However, the observed 102% increase substantially exceeds this demographic contribution. Case detection may be the primary lever due to improvements in several programmatic enhancements during the period. For instance, progressive rollout of GeneXpert (Xpert MTB/RIF) technology since 2011; applied in children using stool, nasopharyngeal aspirate, or induced sputum specimens where expectorated sputum is not obtainable; combined with strengthened pediatric TB clinical algorithms, contributed to improved case detection over the study period [10], [27]. Additionally, external support from the NTLP's partners played a crucial role in providing both technical and direct support to TB case-finding activities [25], [28], [29]. The temporal correlation suggests these efforts were effective in closing the diagnostic gap [3], [4], [30], [31]. This reduction in TB-HIV co-infection is particularly significant for treatment outcomes, given that HIV co-infection accounts for approximately 15% of childhood TB-related mortality [5].

The impact of COVID-19 on childhood TB notifications in Tanzania presents a unique pattern that diverges from global trends. While WHO reported substantial declines in TB case detection globally during 2020 followed by partial recovery in 2021 [1], our data show that notifications in Tanzania remained stable and actually increased throughout the pandemic, with no significant disruption during 2020–2021. This resilience can be attributed to Tanzania's specific COVID-19 response context: unlike many countries, Tanzania did not implement nationwide lockdowns, allowing TB diagnostic and treatment services to continue largely uninterrupted ([32], [33], [34]. The 17% notification decline in 2023 may reflect: (1) depletion of a diagnostic backlog accumulated during 2020–2022; (2) reduction in active case-finding as COVID-related partner support wound down; (3) health worker fatigue following years of dual TB and COVID programming; and (4) possible lag in routine service normalization [25]. Urgent programme review is needed to determine whether this reflects true burden reduction or a surveillance gap.

Moreover, the regional differences reflect disparities in diagnostic infrastructure, human resources, and programme intensity [35], [36]. For instance, Mbeya region has much better infrastructure compared to region Songwe region, however in addition to that, the population in Mbeya region is 74% higher than that in region Songwe in addition to differences in health seeking behavior.

Treatment success in this cohort was high, aligning with recent NTLP reports showing pediatric success rates around 97–98% [27]. These rates surpass global averages (88–92%), suggesting effective treatment delivery, drug supply, and adherence support within the study regions. Comparable high performance has been documented in strengthened TB programmes in Africa [6], [36], [37]. Despite overall success, HIV co-infection was strongly associated with unfavorable outcomes, a finding consistent other studies [1], [5], [38], [39] These results underscore the critical need to close the still available gap in TB-HIV co-infection, integrated TB-HIV care, early ART initiation, and routine provision of TPT and nutritional support. Moreover, younger children (< 5 yrs) were disproportionately associated with unfavorable outcomes. This aligns with biological vulnerability due to immature immunity and the well-documented risk of severe or disseminated TB in younger children [39]. A similar observation was made regarding children referred from inpatient wards possibly because inpatient referral patterns reflect severity, comorbidity or late diagnosis or presentation [40].

This study's primary strengths include analysis of a large national dataset (25,449 cases) providing robust statistical power, comprehensive coverage of all ten high-burden regions, and a six-year observation period enabling trend analysis including COVID-19 impact assessment. These findings are directly relevant to Tanzania's National TB and Leprosy Strategic Plan VI (2020–2025) and the ongoing Xpert MTB/RIF scale-up. The observed gains in case detection underscore continued investment in point-of-care diagnostics, while the 2023 notification decline signals the need for renewed case-finding aligned with NSP VI targets. Rigorous data quality procedures were implemented, and sensitivity analyses demonstrated that exclusion of cases with missing data on key variables did not materially alter findings while maintaining adequate sample size and statistical power.

However, the cross-sectional design limits causal inference, unmeasured confounders could not be evaluated, and low prevalence of unfavorable outcomes (2.8%) restricted multivariable regression, necessitating primarily unadjusted analyses. Despite these constraints, this analysis represents the most comprehensive assessment of childhood TB trends in Tanzania and provides critical evidence for programmatic strengthening. Moreover, age-group-specific TB incidence rates were not calculated due to the unavailability of disaggregated regional population denominators by single-year age group. Additionally, drug susceptibility testing (DST) data were not systematically captured for pediatric cases.

## 5 Conclusion

This study demonstrates significant progress in childhood TB case detection in Tanzania over the 2018–2023 period, with notifications more than doubling. The substantial decline in TB-HIV co-infection, coupled with excellent treatment success rates, is evidence of strong treatment programs for HIV and TB. However, critical vulnerabilities persist: HIV-positive children face nearly three times higher odds of unfavorable outcomes, children under five years remain disproportionately affected by unfavorable outcomes, and marked regional disparities indicate uneven programmatic reach. Most concerning, the 17% decline in notifications from 2022 to 2023 warrants urgent investigation to determine whether it reflects reduced disease burden, weakened case-finding capacity, or reporting lag. Moreover, regional disparities limit the opportunity for the NTLP to consolidate recent gains and accelerate progress toward childhood TB elimination.

## Declaration of generative AI and AI-assisted technologies in the manuscript preparation process

During the preparation of this work, the authors used Claude (Anthropic) to assist with editing and consolidating sections of the manuscript for clarity and conciseness. The authors reviewed and edited all outputs as needed and take full responsibility for the content of the published article.

## CRediT authorship contribution statement

**Evance N.Mgeyi:** Writing – original draft, Validation, Supervision, Resources, Methodology, Investigation, Formal analysis, Conceptualization. **Florence S.Kalabamu:** Writing – review & editing, Validation, Supervision, Methodology, Formal analysis. **Onduru G.Onduru:** Writing – review & editing, Data curation. **Kisonga M.Riziki:** Writing – review & editing, Data curation. **Robert F.Balama:** Writing – review & editing, Data curation. **Galus A.Sililo:** Writing – review & editing, Data curation. **Paul Shimba:** Writing – review & editing. **John Kwesiga:** Writing – review & editing. **Ainembabazi Rose:** Writing – review & editing. **Solomon T.Wafula:** Writing – review & editing, Visualization, Validation, Software, Methodology, Formal analysis, Data curation. **Deo Nsubuga:** Writing – review & editing, Validation, Supervision, Methodology, Formal analysis. **Derrick Kimuli:** Writing – review & editing, Visualization, Validation, Methodology, Formal analysis, Data curation.

## Consent for publication

Not applicable.

## Ethics approval and consent to participate

Administrative clearance was obtained from the Cavendish University School of Postgraduate Studies. Ethical approval was granted by the National Institute for Medical Research (NIMR) through the National Health Research Ethics Committee (NaTHREC) (Reference: NIMR/HQ/R.8a/Vol.IX/4988). As this study involved secondary analysis of de-identified, routinely collected programmatic data, the requirement for individual informed consent was waived by the ethics committee in accordance with national ethical guidelines. Subsequently, permission to utilize programmatic data was obtained from the Tanzania MoH through the NTLP (Reference: GA.154/425/05/111/9). Patient confidentiality and data security were strictly maintained throughout the study. All patient identifiers were removed during data extraction, and only anonymized data were used for analysis. No individual-level identifying information was included in the study findings or publications. This study was conducted in accordance with the Declaration of Helsinki and adhered to the Strengthening of the Reporting of Observational Studies in Epidemiology (STROBE) guidelines for reporting cross-sectional studies.

## Funding

This research did not receive any specific grant from funding agencies in the public, commercial, or not-for-profit sectors.

## Declaration of competing interest

The authors declare that they have no known competing financial interests or personal relationships that could have appeared to influence the work reported in this paper.
