# Supplementary material for: Regional trends in childhood tuberculosis case notification and treatment outcomes: Tanzania, 2018–2023
Source: J Clin Tuberc Other Mycobact Dis. 2026 Jul 30;45:100640. doi: 10.1016/j.jctube.2026.100640 (PMC13452993; doi:10.1016/j.jctube.2026.100640)
Supplement: Supplementary file 1 [file mmc1.docx]

Table S1. Overall Childhood TB case notification trends per region over time

| **Category** | **2018** | **2019** | **2020** | **2021** | **2022** | **2023** | **P -value for trend** | **Direction** |
| --- | --- | --- | --- | --- | --- | --- | --- | --- |
| Overall | 2,688 | 3,404 | 4,536 | 4,907 | 5,421 | 4,493 | 0.047* | Increasing |
| **Region** | | | | | | | | |
| Geita | 486 | 551 | 504 | 531 | 520 | 510 | 0.809 | Increasing |
| Iringa | 181 | 250 | 253 | 223 | 252 | 222 | 0.503 | Increasing |
| Kagera | 360 | 519 | 567 | 586 | 962 | 638 | 0.095 | Increasing |
| Mara | 250 | 411 | 790 | 713 | 879 | 618 | 0.114 | Increasing |
| Mbeya | 279 | 294 | 219 | 182 | 263 | 235 | 0.370 | Decreasing |
| Njombe | 196 | 199 | 173 | 155 | 133 | 256 | 0.843 | Increasing |
| Ruvuma | 189 | 291 | 505 | 726 | 654 | 412 | 0.184 | Increasing |
| Shinyanga | 332 | 465 | 723 | 782 | 659 | 635 | 0.136 | Increasing |
| Songwe | 88 | 81 | 55 | 202 | 177 | 268 | 0.030* | Increasing |
| Tabora | 327 | 343 | 747 | 807 | 922 | 699 | 0.063 | Increasing |
| **Sex** | | | | | | | | |
| Female | 1253 | 1632 | 2142 | 2341 | 2601 | 2165 | 0.040* | Increasing |
| Male | 1435 | 1772 | 2394 | 2566 | 2820 | 2328 | 0.054 | Increasing |
| **Disease classification** | |  |  |  |  |  |  |  |
| Both | 5 | 14 | 32 | 26 | 30 | 44 | 0.010* | Increasing |
| Extra pulmonary | 902 | 1141 | 1589 | 1601 | 1435 | 1262 | 0.284 | Increasing |
| Pulmonary | 1769 | 2249 | 2915 | 3280 | 3965 | 3187 | 0.027 | Increasing |
| **History of Treatment** | | | | | | | | |
| New | 2657 | 3363 | 4496 | 4876 | 5375 | 4469 | 0.045* | Increasing |
| Other | 3 | 9 | 9 | 3 | 6 | 7 | 0.853 | Increasing |
| Relapse | 23 | 23 | 17 | 14 | 22 | 11 | 0.131 | Decreasing |
| LFU | 5 | 9 | 14 | 13 | 18 | 6 | 0.524 | Increasing |
| **HIV status** |  |  |  |  |  |  |  |  |
| HIV Negative | 1922 | 2670 | 3840 | 4380 | 4877 | 4093 | 0.027* | Increasing |
| HIV Positive | 766 | 734 | 696 | 527 | 544 | 400 | 0.002* | Decreasing |

Table S2. Childhood TB case notification trends per region over time among HIV infected

| **Category** | **2018** | **2019** | **2020** | **2021** | **2022** | **2023** | **p–value for trend** |
| --- | --- | --- | --- | --- | --- | --- | --- |
| **Overall** | **766** | **734** | **696** | **527** | **544** | **400** | **0.002** |
| **Region** | | | | | | | |
| Geita | 123 | 103 | 116 | 88 | 91 | 49 | 0.021 |
| Iringa | 62 | 79 | 59 | 44 | 51 | 30 | 0.042 |
| Kagera | 44 | 81 | 48 | 32 | 62 | 30 | 0.438 |
| Mara | 43 | 57 | 47 | 28 | 61 | 34 | 0.679 |
| Mbeya | 120 | 82 | 57 | 38 | 45 | 39 | 0.018 |
| Njombe | 88 | 96 | 45 | 37 | 26 | 38 | 0.031 |
| Ruvuma | 49 | 29 | 35 | 31 | 21 | 20 | 0.024 |
| Shinyanga | 102 | 95 | 121 | 82 | 56 | 50 | 0.050 |
| Songwe | 22 | 16 | 10 | 36 | 15 | 27 | 0.601 |
| Tabora | 113 | 96 | 158 | 111 | 116 | 83 | 0.579 |
| **Sex** |  |  |  |  |  |  |  |
| Female | 359 | 364 | 335 | 249 | 250 | 198 | 0.003 |
| Male | 407 | 370 | 361 | 278 | 294 | 202 | 0.003 |
| **Disease classification** | | | | | | | |
| Both | 0 | 2 | 3 | 1 | 4 | 1 | 0.846 |
| Extra pulmonary | 238 | 238 | 201 | 144 | 129 | 80 | <0.001 |
| Pulmonary | 524 | 494 | 492 | 382 | 411 | 319 | 0.007 |
| **History of Treatment** | | | | | | | |
| New | 747 | 708 | 675 | 519 | 528 | 392 | 0.002 |
| Other | 3 | 5 | 5 | 1 | 4 |  | 0.761 |
| Relapse | 16 | 17 | 4 | 3 | 8 | 6 | 0.131 |
| LTFU |  | 4 | 12 | 4 | 4 | 2 | 0.406 |

Table 2b. Childhood TB case notification trends per region over time among HIV negative children

| **Category** | **2018** | **2019** | **2020** | **2021** | **2022** | **2023** | ***p*–value for trend** |
| --- | --- | --- | --- | --- | --- | --- | --- |
| **Overall** | **1922** | **2670** | **3840** | **4380** | **4877** | **4093** | **0.027** |
| Region | 363 | 448 | 388 | 443 | 429 | 461 | 0.136 |
| Geita | 119 | 171 | 194 | 179 | 201 | 192 | 0.064 |
| Iringa | 316 | 438 | 519 | 554 | 900 | 608 | 0.066 |
| Kagera | 207 | 354 | 743 | 685 | 818 | 584 | 0.104 |
| Mara | 159 | 212 | 162 | 144 | 218 | 196 | 0.534 |
| Mbeya | 108 | 103 | 128 | 118 | 107 | 218 | 0.143 |
| Njombe | 140 | 262 | 470 | 695 | 633 | 392 | 0.160 |
| Ruvuma | 230 | 370 | 602 | 700 | 603 | 585 | 0.069 |
| Shinyanga | 66 | 65 | 45 | 166 | 162 | 241 | 0.018 |
| Songwe | 214 | 247 | 589 | 696 | 806 | 616 | 0.038 |
| Tabora | 894 | 1268 | 1807 | 2092 | 2351 | 1967 | 0.024 |
| **Sex** | | | | | | | |
| Female | 1028 | 1402 | 2033 | 2288 | 2526 | 2126 | 0.029 |
| Male |  |  |  |  |  |  |  |
| **Disease classification** | | | | | | | |
| Both | 5 | 12 | 29 | 25 | 26 | 43 | 0.011 |
| Extra pulmonary | 664 | 903 | 1388 | 1457 | 1306 | 1182 | 0.144 |
| Pulmonary | 1245 | 1755 | 2423 | 2898 | 3545 | 2868 | 0.017 |
| **History of Treatment** | | | | | | | |
| New | 1910 | 2655 | 3821 | 4357 | 4847 | 4077 | 0.026 |
| Other |  | 4 | 4 | 2 | 2 | 7 | 0.613 |
| Relapse | 7 | 6 | 13 | 11 | 14 | 5 | 0.751 |
| LTFU | 5 | 5 | 2 | 9 | 14 | 4 | 0.485 |
